# Case report: atypical presentation of *Mycobacterium tuberculosis* uveitis preceding nodular scleritis

**DOI:** 10.1186/s12879-015-1221-4

**Published:** 2015-10-28

**Authors:** Sunee Chansangpetch, Anita Manassakorn, Prasart Laksanaphuk, Usanee Reinprayoon

**Affiliations:** Department of Ophthalmology, Faculty of Medicine, Chulalongkorn University and King Chulalongkorn Memorial Hospital, 1873 Rama 4 Road, Pathumwan, Bangkok 10330 Thailand

**Keywords:** Mycobacterium tuberculosis, Uveitis, Nodular scleritis, Phacolytic glaucoma, Intraocular inflammation

## Abstract

**Background:**

Intraocular tuberculosis is uncommon and has various clinical presentations. Lack of specific clinical clues can make the diagnosis challenging. The purpose of this study is to report a clinical presentation of tuberculous iridocyclitis that mimics phacolytic glaucoma and has a distinctive inflammatory deposit in the inner side of the cornea. This report is the first to describe the progression of tuberculous iridocyclitis to nodular scleritis without evidence for extraocular tuberculous infection.

**Case presentation:**

A 78-year-old, immunocompetent woman presented with subacute intraocular inflammation with high intraocular pressure, mimicking phacolytic glaucoma. Distinct pigment keratic precipitates were noted on the first visit. Even though the cataract extraction was uneventful and adequate anti-inflammatory drugs were given, the inflammation did not subside as expected. Seven weeks later, she developed two scleral abscesses, which were subsequently explored for microbiological investigation. The smears of the pus revealed positive acid-fast bacilli stain and PCR for Mycobacterium tuberculosis complex. Eventually, the pus culture grew *Mycobacterium tuberculosis*. Anti-tuberculosis medications were prescribed. After 1 month of treatment, the abscesses were cured. However, her visual acuity did not improve at the last visit.

**Conclusions:**

This case revealed an unusual presentation and untreated course of tuberculosis iridocyclitis. Pattern of keratic precipitates may indicate the presence of tuberculosis.

## Background

Most *Mycobacterium tuberculosis* infection involves the lungs which is known as pulmonary tuberculosis. However, tuberculosis can also occur in other places of the body. For example, approximately 15–20 % of immunocompetent patients [1] and 50 % of immunocompromised patients [2] can experience extrapulmonary tuberculosis which can affect the lymphatic system, bone, gastrointestinal system, central nervous system, genitourinary system, cardiovascular system, and the skin.

Another site in which tuberculosis can appear is in the eye but this is rare. The incidence of ocular tuberculosis varies among series of cases. In endemic areas such as Southeast Asia, many immunocompromised HIV patients have extrapulmonary tuberculosis [3]. Usually, the ophthalmic involvement of *Mycobacterium tuberculosis* is secondary from hematogenous spreading. Primary infection is seldomly reported [4] but can occur at many sites such as the orbit, lacrimal gland, eyelid, conjunctiva, sclera, iris, choroid, retina, and optic nerve, and have a broad range of clinical manifestations [4, 5].

Tuberculous infection of the eyes can present with various clinical symptoms and frequently mimic other ophthalmic diseases. Thus, these patients are often misdiagnosed [6]. Many of the challenges of diagnosing tuberculosis in the eye are encountered such as limited amount of specimen available for testing, lack of laboratory investigation, poor yield from intraocular specimen, and the absence of uniformity in its diagnostic criteria [7]. Delayed diagnosis can result in severe visual impairment as well as the loss of the globe.

One of the most common ocular clinical manifestations is the posterior uveitis with choroidal tubercles [5, 6] whereas only 0.6–7.9 % of anterior uveitis or iridocyclitis is caused by tuberculosis [8, 9]. As a result of this, we report the clinical presentation of tuberculous iridocyclitis that mimics phacolytic glaucoma and have distinct deposits in the inner side of the cornea known as keratic precipitates (KP). Moreover, we also describe the clinical progression from uveitis to nodular scleritis in immunocompetent patient which has never been reported elsewhere.

## Case presentation

**Fig. 1:**
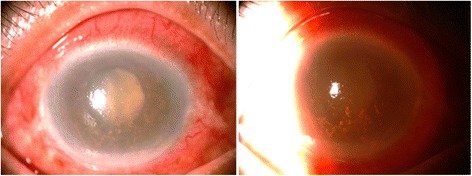
Slit lamp examination of the right eye. (picture shown on the left side) Diffuse illumination showed markedly ciliary injection, corneal edema, and swollen lens. (picture shown on the right side) The sclerotic scatter technique could detect the inferiorly dispersed pigmented keratic precipitates.

A 78-year-old Thai female presented with a right painful ocular irritation and progressive blurring of the vision for 2 months. At the first visit, best-corrected visual acuity was hand motion for the right eye and 20/70 for the left eye. The intraocular pressure was 40 mmHg for the right eye and 10 mmHg for the left eye. On slit lamp examination, her right eye had marked ciliary injection, mild corneal edema with inferior heavily pigmented KP, cells grade 3+, and flares grade 3+ in the anterior chamber. The pupil was mid-dilated, fixed, and did not react to light. The lens was swollen and totally opaque (mature cataract) (Fig. 1). The left eye was unremarkable except for senile immature cataract. She was diagnosed with phacolytic glaucoma of the right eye and underwent extracapsular cataract extraction and intraocular lens (IOL) implantation on the next day without intra-operative complication. In early post-operative period, the intraocular pressure was well controlled without anti-glaucoma medication and the visual acuity slightly improved to 20/200. Topical antibiotic and steroid eye drops were prescribed but the inflammation in the anterior segment of the eye did not subside.

**Fig. 2:**
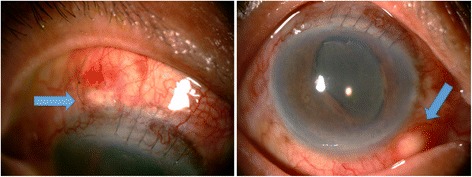
Two yellowish scleral abscesses (indicated by an arrow). An abscess of 2.5 mm in diameter at 11 o’clock, superior to the wound of the extracapsular cataract extraction where the sutures have been removed next to the abscess (in the picture shown on the left side). The other abscess of 2 mm in diameter located at 5 o’clock (in the picture shown on the right side)

Seven weeks later, her right eye developed 2 yellowish scleral masses at 5 and 11 o’clock (Fig. 2). The inflammation continued to persist (cell and flare grade 3+) and fundus could not be examined due to vitreous haze grade 3. Ultrasound of the right eye showed minimal retrolental vitreous opacity and the vitreous echoes showed rapid after movements. The retinal-choroidal thickness was 0.68 mm. Post-operative endophthalmitis was suspected and the patient was sent for vitreous tapping and intravitreous injection with vancomycin and ceftazidime. The culture for aerobic, anaerobic, and fungus revealed all negative results. Topical fortified cefazolin and ceftazidime eye drops were given. When topical steroid was discontinued, this resulted in worsening of the inflammation in the anterior chamber and scleral masses. Because the masses increased in size and showed obvious abscess formation, exploration and debridement of the scleral abscesses were done. Specimen was sent for Gram stain, KOH test, aerobic bacteria, anaerobic bacteria, and fungus cultures, all of which were negative.

**Fig. 3:**
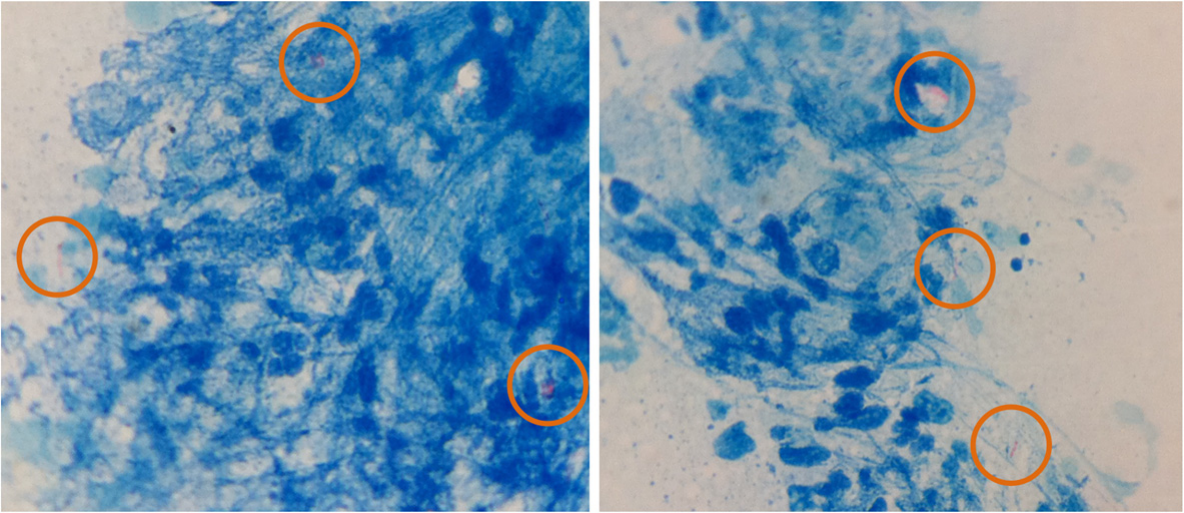
Photomicrograph of the red, short curved, acid fast rod organisms in AFB stain

One week after scleral exploration, her eye was still severely inflamed with corneal edema, cell grade 4+, flare grade 3+, and frank fibrin in front of the IOL. The anterior chamber became shallower and resulted in captured IOL. New scleral abscess was noted at 11 o’clock adjacent to the explored area. The clinical signs were getting worse and did not respond to broad-spectrum fortified antibiotic eye drops. Scleral exploration with abscess debridement was performed for the second time. The authors then became highly suspicious that she may have atypical pathogens so the specimen was also sent for acid-fast bacilli (AFB) and modified AFB stain. The smear showed AFB positive organisms (Fig. 3).

Real-time polymerase chain reaction (PCR) for Mycobacterium tuberculosis complex was done on the scleral abscess, aqueous humor, and vitreous, which were positive in the abscess and aqueous. The tests of the vitreous samplings yielded negative results. Later, the pus culture with a combination of liquid (Mycobacteria growth indicator tube) and solid media (Ogawa) grew *Mycobacterium tuberculosis*.

The patient was referred to an infectious disease specialist to investigate for systemic tuberculosis. She reported a loss of 10 kg of body weight in the past 3 months. She denied fever, night sweats and chest symptoms. Physical examination including her left eye was otherwise normal. The hematocrit was 35.3 % (reference range 36–45 % in women). The white blood cell count was 6500 cells/mm^3^ and the cells were predominantly neutrophils (70 %). The other routine laboratory test results were normal. A chest radiograph revealed normal finding. Sputum AFB was also negative. Neither the evidence of extraocular tuberculosis nor history of tuberculosis contact was identified.

The patient was then started on systemic anti-tuberculosis medications with isoniazid 250 mg/d, rifampicin 450 mg/d, ethambutal 1000 mg/d and pyrazinamide 1250 mg/d. Prednisolone acetate eyedrop was prescribed after starting anti-tuberculosis for a week. A 4-drug anti-tuberculosis regimen was continued for the first 2 months, and for the next 4 months, only isoniazid and rifampicin were used. Regular follow-ups were performed. After 1 month of treatment, no new abscess was found and the intraocular inflammation subsided a bit even though her visual acuity was still hand motion. After 2 months of treatment, the inflammation had completely subsided with no development of new abscess and the IOP was controlled but the vision did not perceive any light so the authors speculated that this was due to the ischemia of the anterior segment.

## Discussion

In our case, at the first visit, she was misdiagnosed as having phacolytic glaucoma because she had a mature cataract and elevated intraocular pressure. However, the persistent post-operative inflammation and progressive scleral abscesses made us reconsider the possibility that the origin of the disease may be infectious, especially an atypical one. Apart from the chronic post-operative endophthalmitis, atypical infectious pathogens such as Nocardia spp. and Mycobacterium spp. initially were included in our differential diagnosis. Endophthalmitis was later ruled out by negative bacteria and fungus culture results. Moreover, the location of vitreous haze was localized only in the retrolental area instead of the vitreous chamber and the normal retinal-choroidal thickness from ultrasonography suggested that the vitreous cells were the reaction to a marked anterior segment inflammation rather than endophthalmitis, a vitreous infection in origin. Our initial differential diagnosis was Nontuberculous mycobacterium because a prolonged steroid use and prior ocular surgery were identified as the risk factors for developing this group of mycobacterium especially the rapid growers [10]. However, when the culture results came back as Mycobacterium tuberculosis, her past clinical course started to make sense.

It should be noted that cataract can frequently develop in the eyes followed by chronic or recurrent uveitis [11]. Severe intraocular inflammation can raise the intraocular pressure due to either the blockage of the intertrabecular space by inflammatory cells or edema of the trabecular meshwork which is the main route for aqueous drainage. The prolonged course of inflammation can permanently damage these meshwork functions. In our case, the patient seemed to have the uveitis for a long period prior to visiting the ophthalmology clinic. These two clinical manifestations, a consequence of a long-run untreated uveitis as aforementioned, misled us to diagnose the patient with phacolytic glaucoma because she had a cataract with high intraocular pressure.

In general phcolytic cases, after cataract surgery, the inflammation should rapidly subside in the first month because the lens, which are the cause of the inflammation, have been removed. Even though the inflammation persisted to 7–8 weeks, it should have been very low grade. However, for this patient, the inflammation subsided a bit in the first week after the cataract surgery when compared to the pre-operative state, but continued to persist at a moderate to high grade throughout the next 7 weeks. Thus, we believe that this inflammation was caused by a pre-existing infection rather than the secondary one.

There are a few cases of intraocular tuberculosis presented with anterior uveitis [6]. Presentation usually includes granuloma of the iris or inflammatory cells at the angle of the anterior chamber previously described as mutton fat KP. Its characteristics are greasy clumps of inflammatory cells at the posterior cornea [6]. This type of KP indicates that it is a granulomatous inflammation, which can also be found in toxoplasmosis and sarcoidosis [10]. However, for this patient, the characteristics of the typical mutton fat KP were absent. Instead, the KPs were heavily pigmented and inferiorly located. The presence of KPs in this patient probably was an important sign that was overlooked which indicated that the etiology was uveitis rather than phacolytic glaucoma. Therefore, we suggest that differential diagnosis of tuberculous uveitis must be kept in mind despite of the absence of a typical mutton fat KP, especially when a prominent pigment is observed.

*Mycobacterium tuberculosis* can also cause scleritis but this is rare [12]. The anterior part of the sclera is affected more frequently than the posterior. Examination may reveal necrotizing nodular scleritis along with scleral ulceration [5]. Kesen, et al. [13] reported, in 2009, a case of atypical drug-resistant tuberculous scleritis presented with masses at the anterior sclera. The masses subsequently progressed to a large area of necrotic sclera. Damodaran, et al. [14] also reported, in 2012, a case with severe intraocular inflammation and a large mass lesion in the globe detected by ultrasonography. The patient did not respond to anti-inflammatory or immunosuppressive agents resulting in perforation of the eye. In both cases, the diagnoses of tuberculous scleritis were confirmed afterward by histopathology of the enucleated eyes.

As far as we are aware, this is the first report that demonstrates the progression of untreated *Mycobacterium tuberculosis* uveitis to scleral abscesses. The progression can be the course of the disease itself when there is a migration of an increasing number of organisms into the adjacent structure, from the intraocular to sclera. It is also possible that the scleral involvement may have been the consequence of a direct inoculation of the organisms during the cataract surgery which could have spread the mycobacterium-filled aqueous to the sclera.

Diagnostic tests for tuberculosis in endemic area are very different compared to non-endemic areas. For example, the tuberculin skin tests and interferon-gamma release assays may be useful for diagnosing tuberculosis in non-endemic area, but the use of these tests are limited and cannot differentiate between the latent tuberculous infection and disease in endemic area such as Thailand [5, 6, 15]. Therefore, in order to diagnose tuberculosis disease in an endemic country, *Mycobacterium tuberculosis* culture, the gold standard, is needed to detect ocular tuberculosis even though the sensitivity of the culture is low for the detection of paucibacillary infection in which case a molecular technique may be helpful bearing in mind the limitation of false-positive results [6].

## Conclusion

In conclusion, this case emphasizes the need to be aware of ocular tuberculosis in an endemic area especially with unusual presentations and prolonged clinical course of ocular inflammation. A high index of suspicion of tuberculosis would require clinical examination aided by an appropriate microbiological testing because it can help in early diagnosis and management of the infection. The pattern of KPs may indicate the presence of tuberculous infection.

## Consent

Written informed consent was obtained from the patient for publication of the case report and any accompanying images. A copy of the written consent is available for review by the Editor of this journal.

## Abbreviations

KP: Keratic precipitate
IOL: Intraocular lens
AFB: Acid-fast bacilli
PCR: Polymerase chain reaction
